# Ovid Discovery

**DOI:** 10.5195/jmla.2020.1089

**Published:** 2020-10-01

**Authors:** Jason A. Lilly, Lisa L. Habegger, Andy Anderson

**Affiliations:** 1 jlilly1@iuhealth.org, Systems Librarian, IU Health Medical Library, Indiana University Health, Indianapolis, IN; 2 lhabegger@iuhealth.org, Manager, Library Services, IU Health Medical Library, Indiana University Health, Indianapolis, IN; 3 janderson27@iuhealth.org, Library Services Coordinator, IU Health Medical Library, Indiana University Health, Indianapolis, IN

## Abstract

**Ovid Discovery.** Ovid Technologies, Global Headquarters, 333 Seventh Avenue, 20th Floor, New York, NY 10001; 800.950.2035; support@ovid.com; https://tools.ovid.com/oviddiscovery/index.html; institutional subscriptions only, contact for pricing.

## INTRODUCTION

Librarians are continually waging a battle against the ease of Google as a one-stop hub for information. Users enter a search phrase and get relevant results generally on the first page, based on the power of the Google algorithm. It is an easy, intuitive process. Librarians attempt to make users understand the power of searching with a controlled vocabulary in subject-specific databases as the initial point for research, but natural language and the simple elegance of the Google search bar keeps this model at the forefront.

Over time, library tools have begun to adapt to this new reality, and Ovid Discovery is a prime example of this phenomenon. A primary feature of Ovid Discovery is the Discovery metasearch, which focuses purely on biomedical sources. The Discovery metasearch pulls relevant results from a library's holdings as well as the Ovid Index, a biomedical database consisting of more than 250 million resources according to the Ovid Discovery site [1]. The reviewers often refer to it as the “One Search to Rule Them All,” which is essentially how it bills itself. In addition to the metasearch, Ovid Discovery serves as a one-stop-shop for a number of library staff processes through its administrative modules. Because the focus is on biomedical resources, the audience for the platform would be hospital, medical, health sciences, or pharmacy libraries. The Indiana University (IU) Health Medical Library has used the Ovid Discovery Service for three years.

## FEATURES

The primary feature of the service is the Discovery metasearch, which is a Google-style search bar that searches across the Ovid Index as well as library holdings. Users enter a search phrase, and results are returned sorted by relevance, much like Google. There are also advanced search options, and the search results page has several filtering options, including classification (article, book, etc.), subject, and date range. If an individual user sets up an account, they can save searches and earn 0.5 continuing medical education (CME) credits for a search run on the platform. Users can also save the search as a really simple syndication (RSS) feed for updated results.

One particularly nice feature of Discovery is that it separates results from other proprietary Wolters Kluwer products like UpToDate and Lippincott Advisor, enabling users to easily jump to these other platforms without having to run a separate search. Individual record pages offer full-text options, if they are available, and provide link outs to Google Scholar to check for open access versions. It is possible to incorporate a service like Unpaywall, which captures individual articles and shows if there is a full-text option available. If an article is not found, a link to the institution's interlibrary loan service is available. There are also standard export options to Word, Excel, portable document format (PDF), plain text, and a variety of reference manager platforms, as well as a citation wizard.

Beyond the Discovery Search, a Library eResources search has a more standard catalog style function, where one can search the library's electronic holdings for journal titles, books, and databases by title, author, and subject, as well as browse journals by title. There is also a Citation Matcher function, where citation information can be added and is matched to records in the Ovid Index and/or library holdings.

## ADMINISTRATIVE MODULES

Ovid Discovery serves as a hub for a number of standard library functions through administrative modules. Once a user is established as an administrator, they can access these modules from a link in the upper right-hand corner of the Discovery site.

### Pages Module

Ovid Discovery can function as a library home page, and its Pages Module serves as a content management system. There are various page templates to choose from, and all are designed to be mobile responsive for optimal viewing on phones or tablets. For page editing, there is a standard what you see is what you get (WYSIWIG) editor for those who are not familiar with hypertext markup language (HTML) coding, while more advanced users can utilize straight HTML in the source code. There are also options for custom cascading style sheets (CSS) that control the design elements to be implemented on the site. Additional features in this module include the ability to create custom forms, surveys, blogs, a basic calendar system, and an RSS feed plugin.

### Reports and Statistics Module

The Statistics Module collects standard website usage statistics and, because it is tied to the Discovery search, offers some unique information. It will provide a breakdown for most-run searches, most-popular articles accessed, and most-accessed journals, as well as statistics on internal pages from the site. In terms of more detailed statistics from individual vendors, there is a Standardized Usage Statistics Harvesting Initiative (SUSHI) interface that allows users to gather reports for journal usage statistics in the Discovery platform. This requires some work in terms of initial setup, but it is nice to have this in one place versus having to log in to each vendor platform.

### Holdings Module

The Holdings Module is another vital part of Ovid Discovery and takes over the cataloging function for electronic resources. This is where all accessible resources are managed including journals, books, and databases. Once standard packages such as ClinicalKey Flex, EBSCO eBooks, and so on are added as part of a library's collection, Discovery does the updating of titles as journals are added and deleted. Individual titles can be added as well and grouped into a la carte collections.

### Security Module

The security protocols are robust and offer a great deal of customization. Based on Internet protocol (IP) authorization rules, Discovery can be locked down to be only accessible to local users or the system can be open to the public. Customization is allowed down to the page level. Global or page-level decisions will need to be made whether to have the site crawled by search engines. With rule sets and content routing, specific groups can tailor the experience on the system to their needs. This is also the area where user accounts are managed and can be elevated to administrator status for access to the back end of the system.

### Additional Modules

Ovid Discovery also functions as a link resolver so when users find articles via various databases, the “Find It” button directly links back to local library holdings for access and not to a third-party solution like Serial Solutions or BrowZine. Settings for this are controlled in the Link Resolver Module. There is also a Document Delivery Module that works well in conjunction with DOCLINE, the National Library of Medicine's automated interlibrary loan system.

Other modules include control settings for the Discovery Search, such as the layout of the results page, and settings for the Library eResources search, as well as the Citation Matcher. Ovid regularly adds features and functionality to the Discovery platform. Information about updates to the product can easily be accessed in the What's New in Ovid DS Module. In terms of usability of the product from the staff side, features and functions are grouped in a logical manner, but in the reviewers' opinion, there is a lot to the product that could be daunting to less tech-savvy users.

## COMPETITION AND CONSIDERATIONS

The head-to-head competition for Ovid Discovery would be EBSCO Stacks Discovery [2]. The reviewers had a demonstration of the Stacks product but really cannot offer a fair comparison having had very little hands-on experience with it. The usability of the Stacks back end interface and the design aspects of templates that were demonstrated were impressive; however, from a budget standpoint, this product was not further considered. The incorporation of other Wolters Kluwer products into Ovid Discovery is a major advantage, especially if these are heavily utilized by an institution.

Content management systems like Drupal, Concrete 5, WordPress, and LibGuides can be used to manage websites. There are advantages to stand-alone systems such as WordPress, based on level of control and flexibility. They offer greater control in terms of back end server-side administration than a proprietary system like Ovid Discovery. They have a robust marketplace of plugins to add features and functionality to sites. With Ovid Discovery, it is necessary to work more closely with their developers if a feature needs to be added.

## GENERAL CONSIDERATIONS

A feature that the reviewers greatly appreciate is the ease with which the Discovery search can pull citations. A title or PubMed identifier (PMID) can be entered, and full-text articles can be quickly accessed, if they are available, with no need to jump to a separate database like PubMed or Google Scholar. The fact that the link-resolver feeds back to the library site is extremely useful. It is a time and mental energy saving for clinicians that can have “butterfly effect” benefits for patients. Librarians put a lot of work into library sites and want them to be useful tools for patrons. While the Discovery metasearch does a decent job with a “quick and dirty search,” the reviewers suggest utilizing subject-specific databases and the power of controlled vocabulary for more complex searching. Ovid Discovery is nice to have in the proverbial tool belt but is not a replacement for all the other resources.

## CONCLUSION

The IU Health System utilizes several Wolters Kluwer products for patient care, education, and research, so the integration of these with Ovid Discovery is a strong selling point. Another advantage for use of this platform is the responsiveness of the Ovid sales and support staff. They resolve problems quickly and provide a high level of customer service. Their head developer has been great to work with, especially as the reviewers prepare to launch into a redesign of the library website. In general, the IU Health Medical Library recommends Ovid Discovery, especially for hospital libraries.
